# Adoption and Use of Social Media in Health Care Among Medical Residents: Cross-Sectional Study

**DOI:** 10.2196/83475

**Published:** 2026-06-05

**Authors:** Zineb Zouakia, Aurélia Manns, Thomas Meyre, Anita Burgun, Natacha Kadlub, Rosy Tsopra

**Affiliations:** 1 Clinical Bioinformatics Laboratory, Imagine Institute, Université Paris Cité, INSERM UMR1163, 24 Boulevard du Montparnasse, Paris, F-75006, France, 33 668169844; 2 Department of Medical Informatics, Hôpital Européen Georges Pompidou, Hôpital Necker Enfants Malades, AP-HP, Paris, France; 3 Department of Maxillofacial Surgery and Plastic Surgery, MAFACE Rare Diseases Reference Centre, Faculty of Medicine, Hôpital Necker Enfants Malades, APHP, Université Paris Cité, Paris, France; 4 Université Paris Cité, UPPERS, US 007, Paris, F-75006, France

**Keywords:** digital health, social media, messaging, WhatsApp, Facebook, health communication, health care professionals, physicians, medical residency, clinical practice, technology acceptance, UTAUT2, Unified Theory of Acceptance and Use of Technology 2

## Abstract

**Background:**

Social media apps are widely used by health care professionals despite security and regulatory risks. Identifying factors associated with this use is important for developing effective risk-reduction strategies.

**Objective:**

This study aimed to investigate how medical residents use 6 popular social media apps in professional tasks and to identify factors influencing their adoption in health care, using the validated Unified Theory of Acceptance and Use of Technology 2 (UTAUT2) model.

**Methods:**

An anonymous web-based survey was conducted between June 2024 and November 2024 among medical residents in France. Participants reported demographic characteristics, frequency, and professional contexts of use for 6 apps (Facebook, Instagram, LinkedIn, Messenger, TikTok, and WhatsApp) and completed UTAUT2-based items. The model was adapted by adding a technology trust construct. Descriptive analyses were performed for all apps. With a sufficient sample size, partial least squares structural equation modeling was conducted for WhatsApp to identify factors associated with behavioral intention and use behavior.

**Results:**

A total of 137 residents (n=87, 63.5% female participants) across 40 specialties completed the survey. WhatsApp was the most widely and professionally used app (n=127, 92.7%), with 75.9% (n=104) using it at least many times per week. It was primarily used for patient care, including written transmissions (n=86, 62.8%), case discussions (n=76, 55.5%), and specialist advice (n=86, 62.8%), as well as for professional networking (n=62, 45.3%). Messenger was used by 46.7% (n=64) of participants for similar purposes. Facebook (n=35, 25.6%) and LinkedIn (n=20, 14.6%) were mainly used for education and networking, whereas Instagram (n=11, 8%) was rarely used, and TikTok was not used for professional purposes. Regarding adoption factors, WhatsApp had the highest overall scores, including the highest performance expectancy (mean 5.4, SD 1.12), behavioral intention (mean 5.28, SD 1.15), and use behavior (mean 5.91, SD 1.30), with high effort expectancy (mean 6.82, SD 0.55) and facilitating conditions (mean 6.07, SD 0.85). LinkedIn showed the highest social influence (mean 5.05, SD 1.06), whereas Instagram showed the highest hedonic motivation (mean 6.61, SD 0.51). Technology trust scores were low across all apps, ranging from a mean of 2.23 (SD 1.16) for Facebook to a mean of 3.72 (SD 1.39) for LinkedIn. In the partial least squares structural equation modeling analysis for WhatsApp, habit was the only significant predictor of behavioral intention (*β*=.53; *P*<.001) and use behavior (*β*=.45; *P*<.001).

**Conclusions:**

WhatsApp dominates professional use among residents despite low trust in its security, and its use is mainly driven by habit. Secure alternatives with features similar to popular social media apps, supported by institutional policies and digital professionalism training, are needed to encourage physicians to better consider safety when using social media.

## Introduction

Social media, including messaging apps, are now common tools in health care [1]. Their use is boosted by the popularity of smartphones and quick communication, especially among young people [2,3]. For example, WhatsApp is used by more than 90% of physicians to share clinical information [4-7]; Facebook is used by more than 70% of health care professionals, primarily for continuing education [8]; LinkedIn is used by more than half for career development [9,10]; and Instagram is popular among the younger users [11], with 68% of medical residents and students using it daily [12]. These apps are used for a wide range of health care activities [1,2,13]: (1) clinical coordination (eg, shift rotation and clinical updates); (2) peer consultation (eg, case discussion and specialist advice); (3) interaction with patients (eg, responding to questions and providing test result updates), with more than 12% of residents having patients (or patients’ family members) listed as social media “friends” [14]; (4) professional networking; and (5) continuing education (eg, access to the latest medical information).

However, the use of such apps raises significant concerns around patient confidentiality and data security, as they fail to comply with data protection standards such as the European General Data Protection Regulation [15] or the US Health Insurance Portability and Accountability Act [16], making them inappropriate for use in health care [17]. To guarantee the security of patient data, secure tools have been developed, such as institutional platforms for patient exchange (eg, intrahospital provider portals; regional or national secure messaging systems [18-20]); private solutions like Siilo [21,22], a secure messaging app for medical professionals; or Pixacare, designed for secure medical photography exchange [23]. Despite the availability of these secure tools, medical professionals continue to prefer using popular social media apps [4], raising questions about the underlying reasons for their continued use.

Several studies have explored the reasons behind the increasing use of social media apps in health care. For example, McGowan et al [24] found that physicians’ use was driven by the ease of use and usefulness in achieving better outcomes, while Nikolic et al [6] pointed out factors such as being free, accessible, widely adopted by peers, and offering features like group chat and data sharing. Additionally, Youssef et al [11] reported that orthopedic surgeons viewed social media as a means to boost visibility, connect with patients, stay informed, and advance their careers. However, prior studies have limitations: (1) they focused on single apps (mostly WhatsApp [7,25-28]) or specific specialties (mostly surgery; eg, orthopedics [11,12,29-32]); (2) they reported general reasons for using social media without examining each app separately [5,6,8,9,12,24,31,33-36]; and (3) they did not use a validated theoretical model or relied on earlier models [24,25,35,37]. For example, the technology acceptance model [38] does not account for relevant factors like habit or social influence.

Here, we aimed to examine the use of social media apps in health care, by investigating how medical residents use 6 popular social media apps in professional contexts, and to identify factors associated with their adoption using the validated Unified Theory of Acceptance and Use of Technology 2 (UTAUT2) model [39].

## Methods

### Study Design

We conducted a cross-sectional study using an anonymous web-based survey to investigate the professional use of social media apps among medical residents and to identify the factors influencing their adoption in health care. The study was designed and reported in accordance with the CHERRIES (Checklist for Reporting Results of Internet E-Surveys) [40] (Checklist 1).

### Participants and Recruitment

Eligible participants were medical residents currently enrolled in postgraduate training programs in France. Participants were recruited between June 25 and November 27, 2024. Recruitment was conducted using a nonprobability convenience sampling strategy. The survey link was disseminated via institutional mailing lists, university networks, and social media through medical residents’ groups. Participants were also encouraged to share the survey with peers, enabling snowball sampling. The recruitment message included a brief description of the study objectives, eligibility criteria, estimated completion time, and a link to the survey. Participation was voluntary, and no incentives were provided.

### Survey Development and Administration

The questionnaire was developed to assess the professional use of social media applications among medical residents and the factors influencing their adoption. It included both descriptive and theory-based components, with the latter based on the UTAUT2 model [39], adapted to the health care context. It was administered online using LimeSurvey and was designed to be completed in approximately 15 minutes. Before dissemination, it was pilot-tested on 3 medical residents to ensure clarity, relevance, and usability.

The survey was structured into 4 sections covering participant characteristics, personal and professional use of social media apps, frequency and contexts of professional use, and factors influencing adoption and use. Participants accessed the survey through a web link and could complete it using a computer or mobile device. They were able to review and modify their responses before submission. No randomization of items was implemented. Adaptive questioning was used based on the apps selected by participants to reduce the number and complexity of questions. To minimize duplicate entries, survey settings were configured to restrict multiple submissions from the same participant.

### Measures

#### Selection of Social Media Apps

The social media apps included in this study were selected based on data from the Digital 2024 Global Overview Report by DataReportal [41], which provides global statistics on digital usage. Apps were chosen according to their number of active users worldwide and to represent different categories. We selected the 3 most widely used multipurpose social media platforms (Facebook, Instagram, and TikTok), the 2 most widely used mobile messaging apps (WhatsApp and Messenger), and the most widely used professional networking platform (LinkedIn). WeChat, although among the most widely used applications globally, was excluded due to its limited use outside of China.

#### Participants’ Characteristics

Participants were asked to provide demographic information, including age, gender, medical specialty, and whether they used each app in their personal lives.

#### Use of Social Media Apps in Health Care

For each app used, participants reported the frequency of use in their professional practice using a 5-point Likert scale (“Many times per day,” “Many times per week,” “Less than once a week,” “Less than once a month,” and “Never”).

Participants also specified the professional contexts in which each app was used. These were assessed using a predefined list of 12 tasks covering 3 domains: patient care (eg, written transmission, case discussion, specialist advice), medical knowledge communication (eg, consumption or creation of educational content), and professional networking (eg, exchange of opportunities, relationship building). The list of tasks was developed empirically based on the authors’ clinical experience as medical residents and doctors, informal discussions with peers, and iterative refinement during questionnaire development. This approach aimed to capture the most common real-world uses of social media apps in clinical practice.

#### Factors of Acceptance and Use in Health Care

To investigate the determinants of social media adoption and use in health care, we used the UTAUT2 model from Venkatesh et al [39], as it is a validated and widely used model for examining factors that influence technology adoption and use [42], including in health care research (eg, telemedicine systems [43] and mobile health apps [44]).

#### Adaptation and Use of the UTAUT2 Model

The original UTAUT2 model includes 7 key constructs (eg, performance expectancy) that predict 2 constructs representing technology use [39]: behavioral intention and use behavior (Table 1). Each construct contains 1 or more items (Table 1).

**Table 1.** Description of the constructs used in our model.

Constructs	Definition	Number of items	Source	Included in model
Performance expectancy	Perceived efficiency gain from using the technology	3	Venkatesh et al [39]	Yes
Effort expectancy	Ease of use	4	Venkatesh et al [39]	Yes
Social influence	Peer influence on the decision to use the technology	3	Venkatesh et al [39]	Yes
Facilitating conditions	Resources and support available to users	4	Venkatesh et al [39]	Yes
Hedonic motivation	Fun or pleasure derived from using the technology	3	Venkatesh et al [39]	Yes
Price value	Perceived value relative to the cost of use	3	Venkatesh et al [39]	No
Habit	Degree to which technology use becomes automatic	3	Venkatesh et al [39]	Yes
Technology trust	Degree to which technology is perceived reliable and secure	3	Casaló et al [45], Yee‐Loong Chong et al [46], Lebrument et al [47]	Yes
Behavioral intention	Intention to use the technology	3	Venkatesh et al [39]	Yes
Use behavior	Actual use (frequency)	1	Venkatesh et al [39]	Yes

For the 7 constructs and behavioral intention, each item is measured using a 7-point Likert scale of agreement (ranging from 1 [strongly disagree] to 7 [strongly agree]), while for use behavior, it is measured using a 7-point Likert scale of frequency of use (from 1 [never] to 7 [many times per day]) [39].

For the purposes of this study, the model was adapted in several ways (Table 1). First, the price value construct was excluded, as all apps assessed were free of charge. Second, a technology trust construct was added to account for perceived reliability and security, which are particularly relevant in the health care context, especially with the sharing of sensitive patient data. This construct and its associated items were added following the guidance of previous studies [45-47].

All items were translated into French based on previously validated translations [48]. While UTAUT2 constructs were measured using 7-point Likert scales, the frequency of use (use behavior) was initially assessed using a 5-point scale for clarity and interpretability. For the statistical analysis, these values were converted to a 7-point scale using linear interpolation to align with the other constructs.

**Figure 1.**
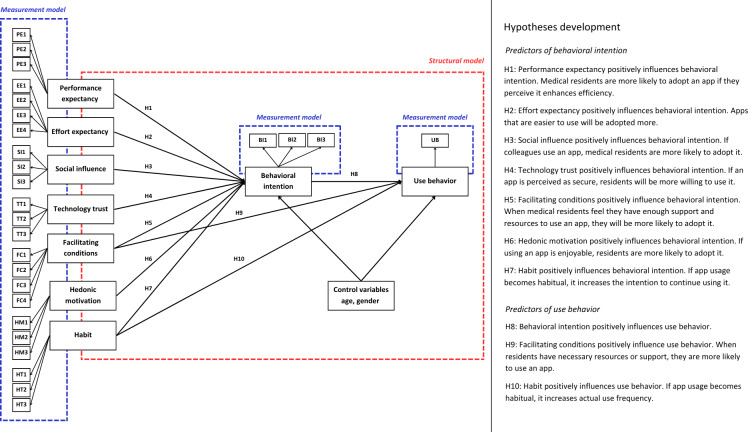
Theoretical framework and hypotheses development of our study based on the Unified Theory of Acceptance and Use of Technology 2 (UTAUT2) model. The measurement model, outlined in blue, illustrates the relationship between the items and their respective constructs, while the structural model, outlined in red, shows the relationships between the predictor constructs and the predicted constructs. BI: behavioral intention; EE: effort expectancy; FC: facilitating conditions; H: hypothesis; HM: hedonic motivation; HT: habit; PE: performance expectancy; SI: social influence; TT: technology trust; UB: use behavior.

Based on the original UTAUT2 hypothesis development [39], we built our model with the following hypotheses: (1) performance expectancy, effort expectancy, social influence, technology trust, facilitating conditions, hedonic motivation, and habit positively influence behavioral intention; (2) behavioral intention, facilitating conditions, and habit positively influence use behavior; (3) age and gender serve as control variables. The final model and its hypotheses are presented in Figure 1.

#### Analysis of the Model According to the Partial Least Squares Structural Equation Modeling Method

To test the model hypotheses, we used partial least squares structural equation modeling (PLS-SEM), a variance-based approach commonly applied in causal modeling [49]. PLS-SEM comprises 2 components: a measurement model, which assesses the relationships between observed items and their corresponding constructs, and a structural model, which evaluates the relationships between constructs and represents the hypothesized paths (Figure 1). Analyses were conducted in RStudio (version 4.3.3; Posit Software, PBC) using the SEMinR package [50,51].

The analysis was performed in 3 steps. First, descriptive statistics were computed to summarize construct scores across apps, using means and SDs.

Second, the measurement model was assessed to evaluate the reliability and validity of the constructs. Prior to analysis, sample size adequacy was verified. The minimum required sample size, based on the number of predictor constructs, was 70 participants [49,52-54]. This threshold was met only for WhatsApp (n=127), leading to the exclusion of Messenger (n=64), Facebook (n=35), LinkedIn (n=20), and Instagram (n=11) from the PLS-SEM analysis. The “Other” gender category (n=2) was excluded due to its very small representation, resulting in a final analytical sample of 125 responses. Measurement model evaluation for WhatsApp included the assessment of internal consistency reliability, convergent validity, and discriminant validity, all of which met the recommended thresholds (Multimedia Appendix 1). Indicator reliability was satisfactory for all items except for 2 items within the facilitating conditions construct. However, they were retained because the construct as a whole demonstrated strong internal consistency and convergent validity, and the items captured distinct, theoretically relevant aspects of the construct, thus maintaining content validity [55].

Third, the structural model was assessed to test the hypothesized relationships between constructs. Collinearity was evaluated using variance inflation factors, which were below the recommended thresholds. Path coefficients (*β*) were estimated using a regression-based approach, and their significance was assessed through bootstrapping with 5000 resamples [49]. The explanatory power of the model was evaluated using the coefficient of determination (*R*²) (Multimedia Appendix 1).

#### Free-Text Comments

In addition to the structured questionnaire items, participants were invited to provide optional free-text comments describing positive and negative perceptions of each app. Comments were analyzed using a thematic approach. Responses were reviewed and grouped into recurring themes. The analysis was conducted by one author (ZZ) and reviewed by another author (RT). Any discrepancies were resolved through discussion until consensus was reached. The frequency of each theme was calculated, and illustrative quotes were selected (Multimedia Appendix 2). These responses were used to complement the quantitative results concerning factors influencing adoption and use (UTAUT2 analysis), providing additional insights into participants’ experiences.

### Statistical Analysis

Only fully completed questionnaires were included in the analysis. Incomplete responses were excluded to ensure data integrity. All statistical analyses were conducted using RStudio (version 4.3.3; Posit Software, PBC). Descriptive statistics were used to summarize participant characteristics, frequency and context of app use, and UTAUT2 construct scores. Categorical variables were reported as counts and percentages, and continuous variables as means and SDs. To investigate factors influencing technology adoption and use, PLS-SEM was conducted for WhatsApp as described earlier. A 2-sided significance level of *P*<.05 was considered statistically significant.

### Ethical Considerations

This study was approved by the CERAP-HP Center Ethics Committee (IRB: IORG0010044). Participants were informed about the study objectives, eligibility criteria, and procedures on the survey’s introductory page. Informed consent was obtained electronically, as completion of the questionnaire implied consent to use anonymized responses for research purposes. No personally identifiable information was collected. Data were anonymous, stored on secure servers, and scheduled for deletion after publication. No financial or material compensation was provided.

## Results

### Participants’ Characteristics

A total of 137 medical residents were included in the study (4 were excluded from the analysis due to inconsistent responses). Most of them were women (n=87, 63.5%) and were aged between 26 and 40 years (n=102, 74.5%). A total of 40 distinct specialties were represented, with pediatrics (n=18, 13.1%), general medicine (n=12, 8.8%), and radiology and medical imaging (n=10, 7.3%) being the most represented (Multimedia Appendix 3).

In private life, the most used apps among the 137 medical residents were WhatsApp (n=133, 97.1%), Messenger (n=132, 96.4%), Facebook (n=120, 87.6%), and Instagram (n=105, 76.6%). LinkedIn (n=10, 7.3%) and TikTok (n=12, 8.8%) were less common.

### Use of Social Media Apps in Health Care

#### Frequency of Use in Health Care

In professional life, the most used app among the 137 medical residents was WhatsApp (n=127, 92.7%). Messenger, Facebook, LinkedIn, and Instagram were used but very less often (n=64, 46.7%; n=35, 25.5%; n=20, 14.6%; n=11, 8%, respectively). TikTok was never used to achieve professional tasks. WhatsApp was used at least “many times per week” by 75.9% (n=104) of residents, while Messenger was used by 32.1% (n=44), Facebook by 10.2% (n=14), Instagram by 4.4% (n=6), and LinkedIn by 3% (n=4; Table 2).

**Table 2.** Frequency of professional use by app (N=137).

Frequency	Facebook, n (%)	Instagram, n (%)	LinkedIn, n (%)	Messenger, n (%)	TikTok, n (%)	WhatsApp, n (%)
Many times per day	3 (2.2)	1 (0.7)	2 (1.5)	15 (11)	0 (0)	64 (46.7)
Many times per week	11 (8)	5 (3.7)	2 (1.5)	29 (21.2)	0 (0)	40 (29.2)
Less than once a week	12 (8.8)	4 (2.9)	8 (5.8)	13 (9.5)	0 (0)	17 (12.4)
Less than once a month	9 (6.6)	1 (0.7)	8 (5.8)	7 (5.1)	0 (0)	6 (4.4)
Never	102 (74.4)	126 (92)	117 (85.4)	73 (53.2)	0 (0)	10 (7.3)

#### Medical Context of Use

The app use depended on context. For patient care, WhatsApp was the most used app among the 137 medical residents, with 62.8% (n=86) of residents using it for written transmission to colleagues and specialist advice, and 55.5% (n=76) for case discussions. Messenger was also used for these purposes but less often. Facebook, Instagram, and LinkedIn were rarely used for clinical tasks.

For medical knowledge communication, Facebook was the top app, mostly for consuming medical training content (n=26, 19.0%). All apps showed minimal use for sharing daily professional life and creating content.

For networking, WhatsApp was the most popular app for exchanging professional opportunities (n=62, 45.3%), followed by Messenger (n=27, 19.7%) and Facebook (n=25, 18.2%). WhatsApp was also the most used app for building professional relationships (n=33, 24.1%), while LinkedIn was used by 12.4% (n=17) of residents for this purpose (Table 3).

**Table 3.** Professional tasks by app (N=137).

Tasks	Facebook, n (%)	Instagram, n (%)	LinkedIn, n (%)	Messenger, n (%)	WhatsApp, n (%)
Patient care					
I make a written transmission to a colleague or another health care professional	2 (1.5)	1 (0.7)	2 (1.5)	32 (23.4)	86 (62.8)
I discuss a patient case with my colleagues or another health care professional	6 (4.4)	2 (1.5)	2 (1.5)	31 (22.6)	76 (55.5)
I request a specialized advice from a colleague or another health care professional	2 (1.5)	2 (1.5)	1 (0.7)	10 (7.3)	86 (62.8)
I discuss a complex case with a multidisciplinary group of experts	3 (2.2)	1 (1.4)	1 (0.7)	2 (1.5)	13 (9.5)
I discuss with a patient about his or her care management	0 (0)	0 (0)	0 (0)	5 (3.7)	12 (8.8)
Medical knowledge communication					
I consume medical training content	26 (19)	11 (8)	8 (5.8)	3 (2.2)	7 (5.1)
I share my daily life as a health care professional with the general public	0 (0)	1 (0.7)	1 (0.7)	4 (2.9)	2 (1.5)
I create training content for health care students	1 (0.7)	0 (0)	1 (0.7)	1 (0.7)	1 (0.7)
I create educational content intended for the general public	0 (0)	1 (0.7)	2 (1.5)	0 (0)	0 (0)
Networking					
I exchange professional opportunities with my peers (job, on-call duties, replacement, courses, etc)	25 (18.2)	4 (2.9)	9 (6.6)	27 (19.7)	62 (45.3)
I build professional relationships	10 (7.3)	3 (2.2)	17 (12.4)	16 (11.7)	33 (24.1)
I share advancements in my specialty with the scientific community or with my colleagues	2 (1.5)	2 (1.5)	4 (2.9)	2 (1.5)	8 (5.8)

### Factors of Acceptance and Use in Health Care

#### Facebook

Facebook scored high in effort expectancy (mean 6.72, SD 0.48) and facilitating conditions (mean 6.11, SD 0.73), indicating ease of use and adequate support. Residents noted its ease of use for sharing information on shift exchanges, medical seminars, and knowledge within medical communities (Ex1, 2, and 4 in Multimedia Appendix 2). However, performance expectancy (mean 3.89, SD 1.53) was moderate, suggesting limited professional effectiveness. Some considered it outdated or inappropriate for professional use, citing misinformation and poor moderation (Ex6-8 in Multimedia Appendix 2). The technology trust score was the lowest among apps (mean 2.23, SD 1.16), reflecting concerns about its security and privacy (Ex10 in Multimedia Appendix 2). Some also criticized it as time-wasting and addictive (Ex9 in Multimedia Appendix 2) (Table 4).

**Table 4.** Scores of the model’s constructs by app. Scores are reported in mean (SD).

	Facebook (n=35), mean (SD)	Instagram (n=11), mean (SD)	LinkedIn (n=20), mean (SD)	Messenger (n=64), mean (SD)	WhatsApp (n=127), mean (SD)
Performance expectancy	3.89 (1.53)	4.09 (1.45)	4.20 (1.30)	4.67 (1.26)	5.43 (1.12)
Effort expectancy	6.72 (0.48)	6.93 (0.16)	5.47 (1.45)	6.84 (0.36)	6.82 (0.55)
Social influence	3.61 (1.11)	3.70 (1.59)	5.05 (1.06)	3.71 (1.19)	4.19 (1.18)
Technology trust	2.23 (1.16)	2.27 (0.88)	3.72 (1.39)	2.41 (1.36)	3.39 (1.43)
Facilitating conditions	6.11 (0.73)	6.45 (0.56)	5.94 (0.76)	6.14 (1.00)	6.07 (0.85)
Hedonic motivation	4.57 (1.46)	6.61 (0.51)	4.48 (1.22)	4.28 (1.38)	4.01 (1.38)
Habit	3.10 (1.29)	2.42 (1.07)	2.80 (1.43)	3.54 (1.43)	3.97 (1.50)
Behavioral intention	4.05 (1.46)	4.39 (1.62)	5.18 (1.17)	4.36 (1.35)	5.28 (1.15)
Use behavior	4.34 (1.41)	4.82 (1.23)	3.85 (1.45)	5.22 (1.39)	5.91 (1.30)

#### Instagram

Instagram had the highest effort expectancy (mean 6.93, SD 0.16) and hedonic motivation (mean 6.61, SD 0.51) among apps, showing that residents found it very easy to use and enjoyed using it for educational content (Ex11 and 12 in Multimedia Appendix 2). However, performance expectancy (mean 4.09, SD 1.45) was moderate, indicating limited professional effectiveness. It was also viewed as addictive, inappropriate for professional use, and lacking relevance (Ex15-17 in Multimedia Appendix 2). The technology trust score (mean 2.27, SD 0.88) was low as well (Table 4).

#### LinkedIn

LinkedIn scored the highest in facilitating conditions (mean 5.94, SD 0.76), indicating adequate support and resources for professional use. Residents noted strengths in networking and career development (Ex19 and 20 in Multimedia Appendix 2). However, performance expectancy was moderate (mean 4.20, SD 1.30), and use behavior was the lowest among apps (mean 3.85, SD 1.45), indicating limited practical use despite its professional focus. Residents confirmed that LinkedIn had little relevance to their daily practice (Ex21 in Multimedia Appendix 2). Technology trust (mean 3.72, SD 1.39) was moderate but the highest among apps. Social influence (mean 5.05, SD 1.06) was also the highest, suggesting adoption was largely peer-driven (Table 4).

#### Messenger

Messenger scored the highest for effort expectancy (mean 6.84, SD 0.36) and facilitating conditions (mean 6.14, SD 1.00), reflecting speed, ease of use, and convenient contact access (Ex24-26 in Multimedia Appendix 2). However, the technology trust score (mean 2.41, SD 1.36) was low because of security concerns (Ex30 in Multimedia Appendix 2). Some residents considered it unsuitable for professional use, citing its link to Facebook, its outdated nature, and missing features found in WhatsApp such as message pinning (Ex28-31 in Multimedia Appendix 2 and Table 4).

#### WhatsApp

##### Average Scores of the Factors Influencing App Use

WhatsApp scored the highest in performance expectancy (mean 5.43, SD 1.12), behavioral intention (mean 5.28, SD 1.15), and use behavior (mean 5.91, SD 1.30) among apps, making it the most frequently used and effective app for professional use. Effort expectancy (mean 6.82, SD 0.55) and facilitating conditions (mean 6.07, SD 0.85) were also high, reflecting ease of use and practical features like phone number integration (Ex32 and 34 in Multimedia Appendix 2). Habit was moderate but the highest among apps (mean 3.97, SD 1.50), indicating WhatsApp’s growing routine use, supported by its popularity and suitability for professional interactions (Ex33 and 37 in Multimedia Appendix 2). Technology trust was also moderate (mean 3.39, SD 1.43) but higher than Facebook, Instagram, and Messenger, with some residents viewing it as relatively more secure despite ongoing privacy concerns (Ex38 and 39 in Multimedia Appendix 2 and Table 4).

##### Factors Influencing Acceptance and Use According to PLS-SEM Analysis

**Figure 2.**
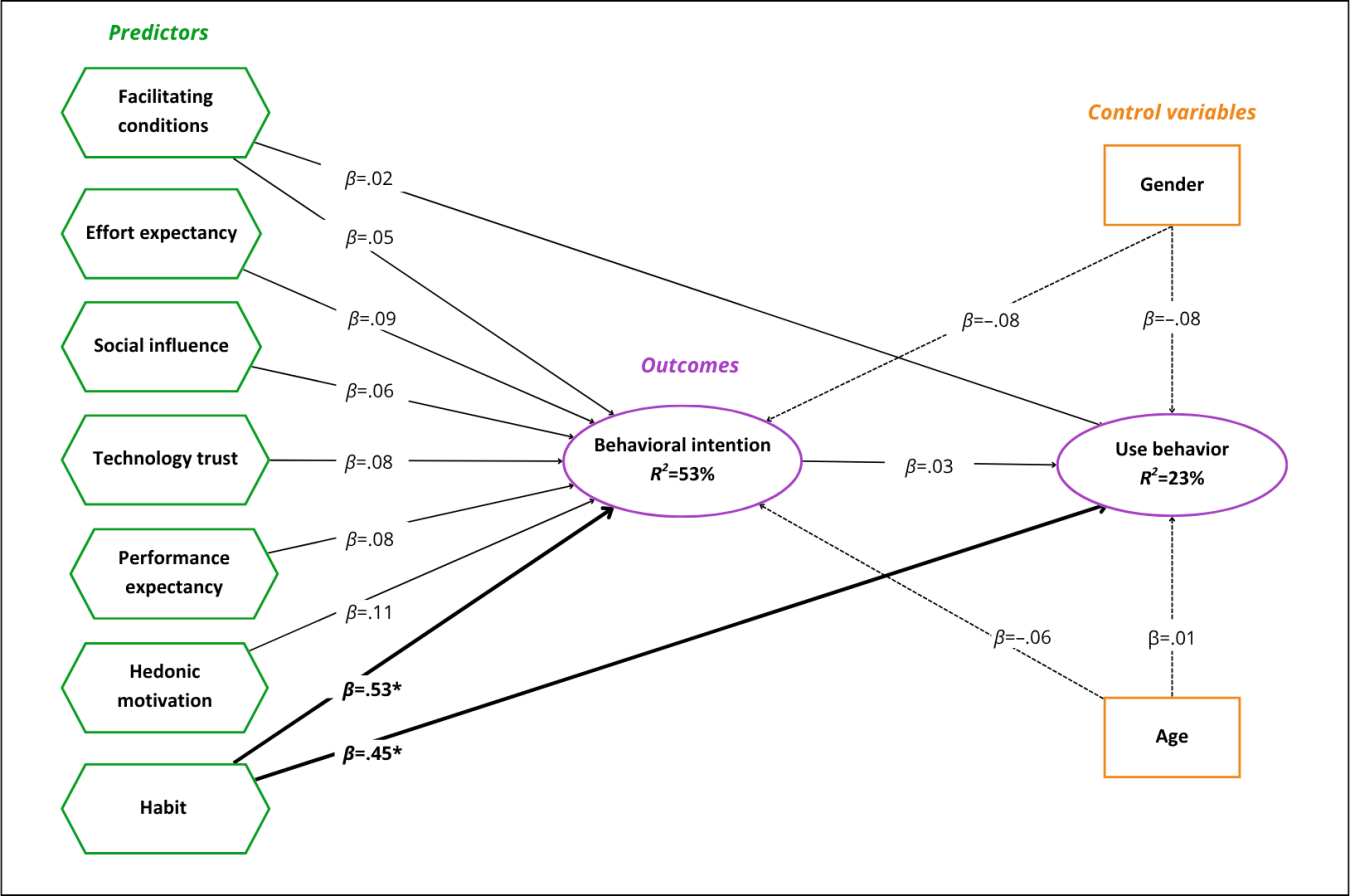
Summary of the structural model results for WhatsApp. The continuous arrows represent the path coefficients (*β*) from the predictor constructs to the predicted constructs; the dotted arrows represent the *β* from the control variables to the predicted constructs; the arrows in bold represent the significant path coefficients; *P<.001; the coefficient of determination (*R*²) represents the explanatory power of the model.

Overall, the model explained 53% of the variance in behavioral intention and 23% of the variance in use behavior (Figure 2). The hypothesized relationships were tested (Table 5, Figure 2). Habit was the only construct with significant effects, showing a positive association with both behavioral intention (H7: *β*=.53, *P*<.001) and use behavior (H10: *β*=.45, *P*<.001).

All other hypothesized paths were not significant. The control variables (age, gender) also showed weak, nonsignificant effects on behavioral intention and use behavior.

**Table 5.** Structural model results for WhatsApp, including path coefficients (*β*), SE, *t* values, and 95% CI for the tested hypotheses.

Path	*β*	*B* ^a^	SE	*t* value	95% CI	Hypotheses (H)
BI^b^						
PE^c^→BI	.08	.09	0.08	1.03	–0.07 to 0.25	H1 (not supported)
EE^d^→BI	.09	.09	0.10	0.88	–0.12 to 0.27	H2 (not supported)
SI^e^→BI	.06	.06	0.07	0.83	–0.08 to 0.20	H3 (not supported)
TT^f^→BI	.08	.08	0.08	0.96	–0.08 to 0.24	H4 (not supported)
FC^g^→BI	.05	.07	0.08	0.65	–0.08 to 0.23	H5 (not supported)
HM^h^→BI	.11	.12	0.08	1.24	–0.05 to 0.28	H6 (not supported)
HT^i^→BI	.53^j^	.52	0.09	5.96	0.34 to 0.69	H7 (supported)
Age→BI	–.06	–.06	0.06	–1.00	–0.18 to 0.07	—^k^
Gender→BI	–.08	–.08	0.06	–1.28	–0.21 to 0.04	—
UB^l^						
BI→UB	.03	.02	0.11	0.31	–0.19 to 0.23	H8 (not supported)
FC→UB	.02	.04	0.13	0.12	–0.22 to 0.29	H9 (not supported)
HT→UB	.45^j^	.45	0.10	4.34	0.24 to 0.65	H10 (supported)
Age→UB	.01	.01	0.09	0.13	–0.15 to 0.18	—
Gender→UB	–.08	–.07	0.08	–0.95	–0.22 to 0.10	—

a *B*: bootstrap path coefficient.

b BI: behavioral intention.

c PE: performance expectancy.

d EE: effort expectancy.

e SI: social influence.

f TT: technology trust.

g FC: facilitating conditions.

h HM: hedonic motivation.

i HT: habit.

j *P*<.001.

k Not applicable.

l UB: use behavior.

## Discussion

### Principal Findings

This study examined the professional use of 6 popular social media platforms among 137 French medical residents across 40 different specialties. Regarding the context of professional use, over 90% of residents reported using WhatsApp professionally, particularly for written transmissions, patient case discussions, and seeking specialist advice. Messenger was used less often for similar purposes. Surprisingly, WhatsApp was also the top app for professional networking, far ahead of LinkedIn, which was used by less than 15% of residents. About 25% of residents used Facebook, mostly as the top app for medical training. Conversely, Instagram was rarely used, mainly for education, and TikTok was never used. Regarding the factors influencing adoption and use, WhatsApp ranked highest overall, leading in performance expectancy, behavioral intention, and use behavior, and showing high effort expectancy, indicating it was perceived as highly effective, easy to use, and likely to remain in use. LinkedIn ranked highest for social influence (peer effect), while Instagram ranked highest for hedonic motivation (entertainment value). Technology trust was low across apps, particularly for Facebook, Instagram, and Messenger, reflecting common security and privacy concerns. Notably, Habit was the only construct significantly associated with both the intention to use and actual use of WhatsApp, suggesting that routine use may outweigh security concerns. These results suggest that informal, noncompliant apps are widely used in clinical practice, mainly because of habit and convenience, even when trust in their security is low.

### Comparison With Prior Work

Regarding the context of professional use, our study found WhatsApp to be the most used app among residents (127/137, 93%), a result that is similar to O’Sullivan et al [7], who reported 100% use among Irish hospital residents. In contrast, Van Ravenswaay et al [8] found Facebook to be the most popular platform among health care professionals (71%) in a survey conducted by the Clinical Education Alliance, based in the United States. In our study, WhatsApp was mainly used for patient care, including transmissions and specialist advice (86/137, 63%) and case discussions (55%), which is similar to the findings in Australia, where 85% of physicians use it for clinical communication (eg, patient management details, imaging reports) [6]. Similarly, Cherrez-Ojeda et al [4] reported a 93% WhatsApp usage rate for physician communication in Ecuador, without detailing clinical contexts. Surprisingly, our study found WhatsApp to be the top app for professional networking, surpassing LinkedIn, which is specifically designed for this purpose. Youssef et al [11] also found that German surgeons preferred messenger apps (34%) over employment platforms (27%). Lastly, our results found limited and mostly passive use of social media for education, consistent with previous studies [8,11,12].

Regarding the factors influencing adoption and use, our study showed that Habit significantly influenced both the intention to use and the actual use of WhatsApp among medical residents, a finding consistent with another study using elements of the technology acceptance model and UTAUT2 [35]. Additionally, other factors such as perceived usefulness [24,35] and ease of use [24] have also been identified as predictors of social media use. However, comparing with other studies is challenging because they differ in terms of studied population (eg, physicians, pharmacists, allied employees [35]); theoretical models used (eg, technology acceptance model [24,25,35], UTAUT2 [35]); or factors examined (eg, habit is not systematically studied [24,25]). The absence of significant effects of other factors in our study may reflect our population: medical residents, a digitally fluent group likely in a late-stage adoption phase, where WhatsApp use is already routine. In such contexts, technology use often becomes automatic, and habit can outweigh the influence of early adoption factors such as perceived usefulness or ease of use, a pattern well documented in postadoption research [56-58].

### Implications for the Future

Our study showed the widespread use of social media in health care, despite security and regulation concerns. Restrictive policies cannot be a solution to prevent security breaches and may even be counterproductive. Instead, various strategies should be adopted at different levels:

IT level: Secure health care apps should be developed with the desirable features of current social media apps while incorporating strong security safeguards. As shown in a prior study [59], required features should include usability (eg, easy and fast), interoperability with electronic health records, safety (eg, secure authentication and secure certified hosting), and functionalities, such as “instant messaging,” “group discussion,” “notification,” or “data storage in electronic health records” [59]. Additionally, the Medical Informatics community has recognized the importance of human factors in the safe and effective adoption of new technologies [60]. As suggested by Kushniruk and Kaufman [61] in 2024, this requires a multilevel perspective, from the individual user to the complex social and organizational context. In parallel, the American Medical Association stressed the need to integrate physicians’ perspectives throughout development so that digital solutions enhance care, fit into workflows, and address concerns such as liability, transparency, and trust [62].

Educational level: Training in digital health, including security, patient confidentiality [63-65], and regulations [66,67], should be implemented in medical curricula [59]. Digital professionalism (ie, responsible use of social media and professional integrity) should be taught as well [68]. Evidence shows that targeted digital professionalism programs improve knowledge of appropriate online behavior, professional conduct, and confidence in privacy and security practices [69]. Innovative approaches, such as interactive workshops reviewing social media content, can lead to meaningful behavior changes [70]. Likewise, promoting critical thinking in digital health [71] and interprofessional team-based training [72] can further strengthen competencies, enhance safety culture, and prepare physicians to practice effectively in a digital health care environment.

Institutional level: Hospitals and health institutions should establish clear, enforceable policies for digital communication, including acceptable tools [59]. They should also provide professional smartphones to separate personal and work use, thereby reducing security risks [3]. Additionally, they should implement and promote the adoption of secure apps, focusing on a bottom-up approach (eg, direct engagement with frontline clinicians and designation of an experienced colleague to lead adoption), which has been proven to increase adoption [73,74]. Finally, institutions should allocate resources to secure communication as part of broader efforts to improve patient safety, efficiency, and regulatory compliance. Financial investment is essential to enable practice change and technology adoption. For example, Sevick et al [75] demonstrated that implementing a digital tool not only improved provider and patient satisfaction but also reduced per-patient costs, illustrating that such investments can be both impactful and cost-effective.

### Limitations

This work has several limitations. First, it focused on French medical residents, which may limit the generalizability to other professional groups, health care systems, or cultural contexts. However, residents are a relevant group as they are heavily involved in clinical work and represent the next generation of physicians likely to use social media in clinical practice. The sample size (N=137) is relatively small, but it spanned 40 specialties, enabling analysis across various disciplines. Due to the sample size, structural modeling (PLS-SEM) could not be performed for all apps [55]. However, we still conducted descriptive factor analysis for each app and used free-text responses to ensure a broad interpretative scope despite the analytical constraints.

Second, reliance on self-reported data may introduce “selection bias,” possibly overrepresenting residents more engaged with social media [76], which may have been further influenced by the use of social media as one of the recruitment channels, potentially favoring more active users. Responses may also be affected by “social desirability bias,” as residents may have described behaviors that do not fully reflect their actual practices due to a desire to appear more responsible or avoid judgment [77]. To limit this bias, the survey was anonymous, which encouraged participants to answer honestly. Likewise, “recall bias” might occur [78], as participants may not remember exactly how they used each app. We mitigated this by designing a structured, detailed questionnaire, breaking down distinct dimensions of use (frequency, professional tasks, and adoption factors), encouraging residents to reflect more precisely on their practices.

Finally, we limited the analysis to 6 adoption factors (performance expectancy, effort expectancy, social influence, facilitating conditions, hedonic motivation, habit), as defined by the validated and widely used UTAUT2 model for technology acceptance and use behavior. We also added a technology trust factor to address health care–specific concerns regarding data privacy and security. However, other factors, such as institutional factors (eg, hospital management policies [25]), could also be considered in further studies.

### Conclusion

This study found that medical residents overwhelmingly used WhatsApp for professional communication, particularly in patient care and networking, with Messenger serving similar functions at a lower frequency. Facebook, Instagram, and LinkedIn were used far less often and mainly for limited purposes, such as education and career development. The analysis of factors influencing adoption and use showed that *habit* was the only significant factor influencing WhatsApp’s use, highlighting the dominance of routine over security concerns. Secure alternatives with similar features to popular social media apps, along with proactive institutional policies and digital professionalism training, are needed to help physicians better consider security when using social media apps.

## Supplementary material

10.2196/83475 Multimedia Appendix 1 The measurement and structural model assessments of the WhatsApp partial least squares structural equation modeling (PLS-SEM) analysis.

10.2196/83475 Multimedia Appendix 2 Free-text comments analysis.

10.2196/83475 Multimedia Appendix 3 Participants’ characteristics.

10.2196/83475 Checklist 1 CHERRIES checklist.

## Abbreviations

CHERRIES: Checklist for Reporting Results of Internet E-Surveys
PLS-SEM: partial least squares structural equation modeling
UTAUT2: Unified Theory of Acceptance and Use of Technology 2
