# Supplementary material for: Adoption and Use of Social Media in Health Care Among Medical Residents: Cross-Sectional Study
Source: JMIR Med Educ. 2026 Jun 5;12:e83475. doi: 10.2196/83475 (PMC13240641; doi:10.2196/83475)
Supplement: Multimedia Appendix 1 [file mededu-v12-e83475-s001.docx]

**Details of the Method section regarding the measurement and structural model assessments of the WhatsApp PLS-SEM analysis**

# **Measurement Model Assessment**

The evaluation of the measurement model followed a 4-steps process:

**Step 1: Indicator reliability assessment**

Indicator reliability refers to how well an individual indicator (item) measures its intended latent variable (construct). It is assessed using the loading of the indicator on its construct, which is simply a correlation between an item and its own construct. To ensure strong indicator reliability, the construct should explain more than half of the variance in the indicators, meaning that ideally, the indicator loadings should exceed 0.70 and indicators below 0.40 should be removed from the measurement model [1].

All loadings were above the 0.70 threshold except for FC3 (slightly under at 0.66) and FC4 (at 0.46) (Table 1). Despite the lower loadings of FC3 and FC4, the overall construct *Facilitating Conditions* demonstrated a strong internal consistency reliability and convergent validity (Table 1). Another factor in deciding whether to remove an indicator is its impact on content validity, referring to how well an indicator captures all aspects of a given construct [2]. Both FC3 and FC4 represent distinct and theoretically relevant dimensions of the construct as defined by the UTAUT2 model [3]. Therefore, both FC3 and FC4 were retained in the measurement model.

**Step 2: Internal consistency reliability assessment**

Internal consistency reliability refers to the degree of correlation between indicators that are intended to measure the same construct and was evaluated through Cronbach alpha (CA) and Composite Reliability (CR) [1]. Both measures exceeded the recommended threshold of 0.7 for all the constructs, indicating internal consistency reliability (Table 1).

**Step 3: Convergent validity assessment**

Convergent validity is the degree to which the construct converges in order to explain the variance of its indicators [1]. The metric used for this evaluation is the average variance extracted (AVE) for all indicators on each construct. An AVE of 0.5 or higher is considered acceptable, as it indicates that the construct explains 50 percent or more of the variance of the indicators that define it [2]. All the constructs had an AVE above 0.5, demonstrating convergent validity (Table 1).

**Table 1.** Indicator reliability, internal consistency reliability and convergent validity of the measurement model (^a^PE: performance expectancy, ^b^EE: effort expectancy, ^c^SI: social influence, ^d^TT: technology trust, ^e^FC: facilitating conditions, ^f^HM: hedonic motivation, ^g^HT: habit , ^h^BI: behavioral intention, ^i^CA: Cronbach alpha, ^j^CR: composite reliability  ^k^AVE: average variance extracted, ^l^Numbers in italic represent the loadings of the indicators for their respective construct. Other indicator numbers represent their cross loadings for the other constructs)

| Construct | PE^a^ | EE^b^ | SI^c^ | TT^d^ | FC^e^ | HM^f^ | HT^g^ | BI^h^ | CA^i^ | CR^j^ | AVE^k^ |
| --- | --- | --- | --- | --- | --- | --- | --- | --- | --- | --- | --- |
| **PE** |  |  |  |  |  |  |  |  | 0.84 | 0.90 | 0.75 |
| PE1 | *0.86^l^* | 0.39 | 0.25 | 0.22 | 0.16 | 0.13 | 0.57 | 0.50 |  |  |  |
| PE2 | *0.89* | 0.35 | 0.19 | 0.19 | 0.21 | 0.25 | 0.48 | 0.39 |  |  |  |
| PE3 | *0.86* | 0.35 | 0.15 | 0.23 | 0.20 | 0.21 | 0.43 | 0.36 |  |  |  |
| **EE** |  |  |  |  |  |  |  |  | 0.96 | 0.97 | 0.88 |
| EE1 | 0.41 | *0.92* | 0.01 | 0.16 | 0.42 | 0.22 | 0.30 | 0.36 |  |  |  |
| EE2 | 0.38 | *0.95* | 0.11 | 0.19 | 0.41 | 0.20 | 0.30 | 0.29 |  |  |  |
| EE3 | 0.35 | *0.95* | 0.14 | 0.23 | 0.43 | 0.27 | 0.31 | 0.37 |  |  |  |
| EE4 | 0.44 | *0.94* | 0.11 | 0.22 | 0.43 | 0.27 | 0.31 | 0.37 |  |  |  |
| **SI** |  |  |  |  |  |  |  |  | 0.90 | 0.94 | 0.83 |
| SI1 | 0.21 | 0.10 | *0.92* | 0.20 | -0.05 | 0.02 | 0.37 | 0.34 |  |  |  |
| SI2 | 0.18 | 0.09 | *0.92* | 0.20 | -0.02 | -0.02 | 0.39 | 0.27 |  |  |  |
| SI3 | 0.24 | 0.07 | *0.89* | 0.24 | -0.03 | 0.03 | 0.33 | 0.27 |  |  |  |
| **TT** |  |  |  |  |  |  |  |  | 0.83 | 0.90 | 0.74 |
| TT1 | 0.15 | 0.16 | 0.21 | *0.88* | 0.18 | 0.34 | 0.21 | 0.24 |  |  |  |
| TT2 | 0.28 | 0.25 | 0.18 | *0.85* | 0.21 | 0.35 | 0.29 | 0.37 |  |  |  |
| TT3 | 0.18 | 0.12 | 0.22 | *0.85* | 0.13 | 0.26 | 0.33 | 0.31 |  |  |  |
| **FC** |  |  |  |  |  |  |  |  | 0.73 | 0.83 | 0.56 |
| FC1 | 0.25 | 0.46 | -0.08 | 0.17 | *0.90* | 0.13 | 0.19 | 0.23 |  |  |  |
| FC2 | 0.22 | 0.40 | -0.04 | 0.08 | *0.88* | 0.14 | 0.12 | 0.15 |  |  |  |
| FC3 | 0.03 | 0.35 | -0.07 | 0.11 | *0.66* | 0.05 | -0.06 | 0.06 |  |  |  |
| FC4 | 0.00 | 0.08 | 0.10 | 0.22 | *0.46* | 0.27 | 0.10 | 0.18 |  |  |  |
| **HM** |  |  |  |  |  |  |  |  | 0.86 | 0.90 | 0.75 |
| HM1 | 0.13 | 0.15 | -0.03 | 0.39 | 0.22 | *0.85* | 0.06 | 0.16 |  |  |  |
| HM2 | 0.27 | 0.29 | 0.04 | 0.29 | 0.17 | *0.92* | 0.27 | 0.35 |  |  |  |
| HM3 | 0.09 | 0.14 | -0.03 | 0.36 | 0.16 | *0.82* | 0.08 | 0.15 |  |  |  |
| **HT** |  |  |  |  |  |  |  |  | 0.72 | 0.84 | 0.64 |
| HT1 | 0.61 | 0.43 | 0.30 | 0.29 | 0.22 | 0.18 | *0.86* | 0.62 |  |  |  |
| HT2 | 0.41 | 0.15 | 0.34 | 0.33 | 0.14 | 0.28 | *0.82* | 0.55 |  |  |  |
| HT3 | 0.32 | 0.07 | 0.33 | 0.14 | 0.01 | -0.03 | *0.71* | 0.42 |  |  |  |
| **BI** |  |  |  |  |  |  |  |  | 0.82 | 0.89 | 0.73 |
| BI1 | 0.52 | 0.40 | 0.27 | 0.24 | 0.23 | 0.19 | 0.59 | *0.87* |  |  |  |
| BI2 | 0.27 | 0.18 | 0.33 | 0.44 | 0.16 | 0.31 | 0.55 | *0.80* |  |  |  |
| BI3 | 0.46 | 0.33 | 0.23 | 0.26 | 0.21 | 0.25 | 0.59 | *0.90* |  |  |  |

**Step 4 : Discriminant validity assessment**

Discriminant validity indicates the extent to which a construct is empirically distinct from other constructs in the model [1]. The first approach was through cross-loadings. An indicator's loading on its associated construct should be greater than its loadings on any other constructs (cross-loadings), as confirmed in Table 1 [1]. Heterotrait–monotrait ratio (HTMT) of correlations [4] is also recommended, by comparing the correlations between different constructs (heterotrait) to the correlations within the same construct (monotrait). HTMT values should be below 0.90 or 0.85. This condition was met in our case, indicating discriminant validity of the model (Table 2).

**Table 2.** Discriminant validity of the measurement model (Heterotrait-Monotrait ratio) (^a^PE: performance expectancy, ^b^EE: effort expectancy, ^c^SI: social influence, ^d^TT: technology trust, ^e^FC: facilitating conditions, ^f^HM: hedonic motivation, ^g^HT: habit , ^h^BI: behavioral intention, ^i^UB: Use Behavior)

| Construct | PE^a^ | EE^b^ | SI^c^ | TT^d^ | FC^e^ | HM^f^ | HT^g^ | BI^h^ | Age | Gender | UB^i^ |
| --- | --- | --- | --- | --- | --- | --- | --- | --- | --- | --- | --- |
| PE |  |  |  |  |  |  |  |  |  |  |  |
| EE | 0.46 |  |  |  |  |  |  |  |  |  |  |
| SI | 0.26 | 0.10 |  |  |  |  |  |  |  |  |  |
| TT | 0.28 | 0.23 | 0.27 |  |  |  |  |  |  |  |  |
| FC | 0.22 | 0.52 | 0.12 | 0.26 |  |  |  |  |  |  |  |
| HM | 0.23 | 0.24 | 0.06 | 0.46 | 0.26 |  |  |  |  |  |  |
| HT | 0.70 | 0.32 | 0.50 | 0.40 | 0.25 | 0.27 |  |  |  |  |  |
| BI | 0.57 | 0.40 | 0.38 | 0.43 | 0.27 | 0.30 | 0.86 |  |  |  |  |
| Age | 0.03 | 0.04 | 0.04 | 0.11 | 0.11 | 0.12 | 0.10 | 0.03 |  |  |  |
| Gender | 0.11 | 0.02 | 0.13 | 0.08 | 0.19 | 0.09 | 0.07 | 0.07 | 0.00 |  |  |
| UB | 0.44 | 0.27 | 0.26 | 0.21 | 0.11 | 0.06 | 0.53 | 0.38 | 0.05 | 0.06 |  |

# **Structural Model Assessment**

Structural model assessment followed a 3-steps process:

**Step 1: Collinearity issues assessment**

Structural model path coefficients are derived from regression equations, but strong correlations between constructs can bias results. Therefore it’s important to check for collinearity, by calculating the Variance Inflation Factor (VIF), with values above 5 indicating potential collinearity issues [1]. All VIF values were below the 5 threshold, ranging from 1.03 to 1.94, confirming the absence of collinearity.

**Step 2: Significance of the paths assessment**

The structural model path significance levels were estimated using a bootstrapping method with 5000 iterations of resampling, to estimate standard errors and compute confidence intervals, as recommended [2]. A path coefficient is considered statistically significant at the 5% level if its 95% confidence interval does not include zero. The significance of the path coefficients are shown in Table 5 of the article.

**Step 3: Explanatory power assessment**

This step involves examining the coefficient of determination (R^2^) of the constructs, which indicates how much of the variance in an predicted variable is explained by the model’s constructs. Overall, our model explained 53% of the variance for *Behavioral Intention* and 23% of the variance for *Use Behavior*.

# **References**

1. Hair JF, Hult GTM, Ringle CM, Sarstedt M. A Primer on Partial Least Squares Structural Equation Modeling (PLS-SEM). Second edition. SAGE; 2017. ISBN: 9781483377452

2. Hair JF, Hult GTM, Ringle CM, Sarstedt M. A Primer on Partial Least Squares Structural Equation Modeling (PLS-SEM). Third edition. SAGE Publications, Incorporated; 2022. ISBN: 9781544396422

3. Venkatesh, Thong, Xu. Consumer Acceptance and Use of Information Technology: Extending the Unified Theory of Acceptance and Use of Technology. MIS Q. 2012;36(1):157. doi:10.2307/41410412

4. Henseler J, Ringle CM, Sarstedt M. A new criterion for assessing discriminant validity in variance-based structural equation modeling. J Acad Mark Sci. 2015;43(1):115-135. doi:10.1007/s11747-014-0403-8
