# Supplementary material for: Adoption and Use of Social Media in Health Care Among Medical Residents: Cross-Sectional Study
Source: JMIR Med Educ. 2026 Jun 5;12:e83475. doi: 10.2196/83475 (PMC13240641; doi:10.2196/83475)
Supplement: Multimedia Appendix 2 [file mededu-v12-e83475-s002.docx]

**Free-text comments analysis**

**Table 1.** Positive and negative feedback on the use of each app in healthcare (Ex: Example of free-text responses)

| App | Feedback type | Theme | Frequency | Examples of free-text responses |
| --- | --- | --- | --- | --- |
| Facebook | *Positive* | **Communication**  Information | 9 | Ex1: “Information on shift exchanges, exchange programs, leave of absence, taxes”, “Stay informed about seminars, conferences, etc., in my field”, “Useful for sharing information within class groups” |
|  |  | Medical Community (Groups and pages) | 8 | Ex2: “Possibility to create groups bringing together a large number of people around different topics, richness of discussions in these groups, knowledge sharing”, “Big community”, “Information pages on a specific field” |
|  |  | Content | 4 | Ex3: “Medical journal content production”, “Image” |
|  |  | **Ergonomics**  Ease of use | 4 | Ex4: “I appreciate the ease of use”, “Easy for…”, “Simple for…” |
|  |  | **Networking**  Social Connections | 3 | Ex5: “University friends who pursued other specialties, making it easier to get advice”, “Stay connected with friends/acquaintances from a distance” |
|  | *Negative* | **Communication**  Obsolescence and lack of relevance | 7 | Ex6: “Is becoming increasingly archaic”, “Has-been”, “Uninteresting”, “Not very useful” |
|  |  | Inappropriate for professional use | 2 | Ex7: “Not suited for medical use, more for personal use” |
|  |  | **Ergonomics**  Poor visibility and misinformation | 7 | Ex8: “Poor visibility of information”, “Lots of misinformation”, “fake news”, “Not enough moderation”, “Lots of ads” |
|  |  | Time-wasting and addictive | 3 | Ex9: “Addictive”, “Time-consuming”, “Waste of time” |
|  |  | **Security and privacy** | 4 | Ex10: “Lack of privacy”, “Not secure”, “Not knowing where the information is being shared in public groups” |
| Instagram | *Positive* | **Communication**  Entertaining content | 6 | Ex11: “Entertainment”, “Entertaining content”, “Leisure, simple escape” |
|  |  | Educational content (professional and public audiences) | 5 | Ex12: “Useful for medical popularization”, “Creative, educational, entertaining content. Allows for discovering and retaining information in a more engaging way” |
|  |  | Content | 3 | Ex13: “Video”, “Photo”, “Image” |
|  |  | **Networking**  Social Connections | 3 | Ex14: “People I follow”, “Small circle of friends”, “Possibility to put a face to a name” |
|  | *Negative* | **Communication**  Inappropriate for professional use | 6 | Ex15: “Not suited for medical use, but sometimes useful for training purposes”, “No medical interest, mainly photos”, “Personal network” |
|  |  | Lack of relevance | 4 | Ex16: “No interest in my opinion (fake and made to show off)”, “I don't find the content serious enough” |
|  |  | **Ergonomics**  Time-wasting and addictive | 3 | Ex17: “Too time-consuming”, “Entertaining but addictive” |
|  |  | **Security and privacy** | 1 | Ex18: “Lack of privacy” |
| LinkedIn | *Positive* | **Networking**  Professional Networking | 6 | Ex19: “Connecting with people from diverse backgrounds in a much more professional setting than other networks, with the ability to easily make contact and exchange ideas on careers and practices” |
|  |  | Career and Professional Development | 4 | Ex20: “Career information, conference,specialization”, “Quite useful for staying updated on scientific advancements in certain fields” |
|  | *Negative* | **Communication**  Lack of relevance | 13 | Ex21: “Not very suited for medical residents, little use” |
|  |  | Excessive self-promotion and marketing | 1 | Ex22: “Lots of storytelling and posts from people who want to promote their business” |
|  |  | **Ergonomics**  Unclear or confusing interface | 1 | Ex23: “Obscure interface” |
| Messenger | *Positive* | **Ergonomics**  Speed and ease of use | 17 | Ex24: “Easy and instant exchange”, “Speed”, “Ease of use”, “Simple communication” |
|  |  | Features and accessibility | 6 | Ex25: “Allows for group conversations”, “Accessible to all”, “Convenient because it's linked to Facebook” |
|  |  | Easy access to contacts | 4 | Ex26: “Quickly find the contact of colleagues whose personal phone number you don't have”, “Contact often available by name” |
|  |  | **Communication**  Information (mostly coworker organization) | 8 | Ex27: “Group with co-residents, information, scheduling, organization”, “Practical for scheduling”, “To discuss a case with my externship friends”, “Exchange with the residents of my subdivision” |
|  | *Negative* | **Communication**  Inappropriate for professional use | 7 | Ex28: “Widely used messaging, but linked to Facebook, more personal than WhatsApp”, “Affiliated with a Facebook account, so difficult to use outside of a personal/family context” |
|  |  | Obsolescence | 1 | Ex29: “A little old” |
|  |  | **Security and privacy** | 4 | Ex30: “Lack of security, frequent hacking”, “Personal data not secure enough, in my opinion” |
|  |  | **Ergonomics**  Missing features | 1 | Ex31: “Lacks some features that WhatsApp has, like pinning information/messages” |
| WhatsApp | *Positive* | **Ergonomics**  Speed and ease of use | 23 | Ex32: “Instantaneity, ease of use”, “Practical and fast communication” |
|  |  | Popularity and wide adoption | 11 | Ex33: “Nearly universal app”, “Required by work”, “Everyone uses it, so it's very practical...” |
|  |  | Features and accessibility | 9 | Ex34: “Doesn't require much network, so it works in the hospital, and it's possible to pin messages”, “Linked to the phone number, no need to create an account on a social network, just need a phone” |
|  |  | **Communication**  Information (mostly advice seeking, patient care) | 19 | Ex35: “We use WhatsApp to receive images in order to provide neurosurgical advice to doctors across the Aquitaine region”, “Discussion group with the supervisors, daily matters of the operating room and department, avoids multiplying calls with the DECT” , “Interact with colleagues for external doctors requesting advice” |
|  |  | Content | 9 | Ex36: “Sending media (e.g., CT scan videos, photos of scars) for advice”, “Good photo/video quality for advice”, “Easily send images (X-ray, ECG)” |
|  |  | More appropriate for professional use | 3 | Ex37: “Respect for privacy, better separation of the professional sphere by only communicating via WhatsApp and not through other platforms with a more 'private' profile”, “Not affiliated with a Facebook account, so it allows for use with less familiar people” |
|  |  | **Security and privacy** | 3 | Ex38: “Efficient and secure”, “More secure than the other apps” |
|  | *Negative* | **Security and privacy** | 5 | Ex39: “Since this app is not secure, it still raises a privacy issue regarding data confidentiality”,  “Data security problem: since we're having a private conversation, we may “forget” that the messaging system isn't secure, and therefore enter information that could lift anonymity... so vigilance is required” |
| Other |  | **Other apps** |  | Ex40: “I've had the chance to try other apps specifically designed to secure data, but they never worked properly, and we always ended up going back to widely used public apps” |
|  |  | **Productivity** |  | Ex41: “On the 'productivity improvement' side thanks to instant messaging and other networks: I find it difficult to know if we're truly gaining productivity from them because we've never functioned without them...” |
