# Supplementary material for: Adoption and Use of Social Media in Health Care Among Medical Residents: Cross-Sectional Study
Source: JMIR Med Educ. 2026 Jun 5;12:e83475. doi: 10.2196/83475 (PMC13240641; doi:10.2196/83475)
Supplement: Multimedia Appendix 3 [file mededu-v12-e83475-s003.docx]

**Participant’s characteristics**

**Table 1.** Participant’s characteristics (N=137).

|  | Overall (N=137), n(%) |
| --- | --- |
| **Age** |  |
| 20-25 years | 35 (25.5%) |
| 26-40 years | 102 (74.5%) |
| **Gender** |  |
| Female | 87 (63.5%) |
| Male | 48 (35.0%) |
| Other | 2 (1.5%) |
| **Specialty** |  |
| Pediatrics | 18 (13.1%) |
| General medicine | 12 (8.8%) |
| Radiology and medical imaging | 10 (7.3%) |
| Psychiatry | 8 (5.8%) |
| Public health | 6 (4.4%) |
| Internal medicine and clinical immunology | 5 (3.6%) |
| Geriatrics | 5 (3.6%) |
| Emergency medicine | 5 (3.6%) |
| Medical biology | 5 (3.6%) |
| Other (Specialties with n < 5 participants, e.g., Anesthesia-intensive care, Neurology, Visceral and digestive surgery, Endocrinology, Orthopedic and trauma surgery, Gynecology) | 63 (50.0%) |
