# Supplementary material for: Adoption and Use of Social Media in Health Care Among Medical Residents: Cross-Sectional Study
Source: JMIR Med Educ. 2026 Jun 5;12:e83475. doi: 10.2196/83475 (PMC13240641; doi:10.2196/83475)
Supplement: Checklist 1 [file mededu-v12-e83475-s004.docx]

| 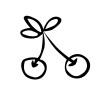 | **Checklist for Reporting Results of Internet E-Surveys (CHERRIES)** | |
| --- | --- | --- |
| ***Item Category*** | ***Checklist Item*** | ***Explanation*** |
| **Design** | Describe survey design | Cross-sectional anonymous web-based survey of medical residents in postgraduate training in France. The sample was a non-probability convenience sample recruited via institutional mailing lists, university networks, and social media, with additional snowball sampling. |
|  |  |  |
| **IRB (Institutional Review Board) approval and informed consent process** |  |  |
|  | IRB approval | The study was approved by the CERAP-HP Centre Ethics Committee (IRB: IORG0010044). |
|  | Informed consent | Participants were informed about the study on the survey’s introductory page before accessing the questionnaire. This page provided details on the study objectives, eligibility criteria, procedures, and the estimated completion time (approximately 15 minutes). The investigators introduced themselves, and provided contact email addresses for participants to reach them if needed. Participants were informed that no personally identifiable information would be collected, that responses would be anonymous, stored on secure servers, and deleted after publication. Informed consent was obtained electronically, as completion of the questionnaire implied consent to participate and to use anonymized data for research purposes. |
|  | Data protection | No personally identifiable information was collected in this study. All responses were fully anonymous. Data were collected and stored using LimeSurvey through an institutional account, ensuring secure hosting and restricted access. Data were stored on secure servers and scheduled for deletion after publication. |
| **Development and pre-testing** |  |  |
|  | Development and testing | The survey was developed based on the literature and established theoretical frameworks, particularly the UTAUT2 model, and adapted to the healthcare context (see Methods section). It included both descriptive and theory-based components. Before deployment, the survey was pilot tested on 3 medical residents to evaluate clarity, relevance, usability, and technical functionality. Feedback from this testing phase informed final revisions of the survey. |
| **Recruitment process and description of the sample having access to the questionnaire** |  |  |
|  | Open survey versus closed survey | Open survey (accessible via a web link). |
|  | Contact mode | The survey link was distributed via institutional mailing lists, university networks, and social media apps (medical residents groups). |
|  | Advertising the survey | The survey was advertised through multiple channels, including institutional mailing lists, university networks, and social media apps. The recruitment message provided a brief description of the study objectives, eligibility criteria, estimated completion time, and a link to the survey. Participants were also encouraged to share the survey with peers, enabling additional dissemination through snowball sampling. |
| **Survey administration** |  |  |
|  | Web/E-mail | Web-based survey administered using LimeSurvey. |
|  | Context | The survey was disseminated via institutional mailing lists, university networks, and social media groups targeting medical residents, which are primarily used for academic and professional communication. This may have led to a sample biased toward residents more digitally engaged, potentially influencing reported use and perceptions of social media in healthcare. |
|  | Mandatory/voluntary | Participation was voluntary. |
|  | Incentives | No incentives were provided. |
|  | Time/Date | The survey was conducted between June 25 and November 27, 2024. |
|  | Randomization of items or questionnaires | No randomization of items or questionnaires was implemented, as the survey followed a structured and logical sequence aligned with the study objectives. |
|  | Adaptive questioning | Adaptive questioning was used, where certain items were displayed conditionally based on participants’ responses (e.g., depending on the social media apps selected), in order to reduce the number and complexity of questions. |
|  | Number of Items | The questionnaire consisted of 4 sections: (1) demographic characteristics (4 items: age, gender, specialty, and use of social media in private life); (2) professional use of social media (2 main items assessing frequency of use and contexts of use, the latter including 12 predefined sub-items describing different professional situations); (3) factors influencing adoption and use based on the UTAUT2 model (8 constructs, each measured by 3 to 4 items); (4) an optional free-text comment section. |
|  | Number of screens (pages) | The questionnaire was divided into several screens corresponding to its 4 main sections (demographics, professional use, UTAUT2 constructs, and free-text comments). |
|  | Completeness check | A completeness check was implemented after submission, as only fully completed questionnaires were included in the analysis. Participants were required to answer all mandatory items before submitting the survey, while non-response options (e.g., “not applicable”) were available where appropriate. |
|  | Review step | Participants were able to review and modify their responses before submission using the survey interface. |
| **Response rates** |  |  |
|  | Unique site visitor | Not assessed, as view and participation rates were not calculated in this study. |
|  | View rate (Ratio of unique survey visitors/unique site visitors) | Not assessed, as the number of unique site visitors and unique survey visitors was not tracked. |
|  | Participation rate (Ratio of unique visitors who agreed to participate/unique first survey page visitors) | Not assessed, as the number of unique visitors to the first survey page and those who agreed to participate was not tracked. |
|  | Completion rate (Ratio of users who finished the survey/users who agreed to participate) | Not assessed, as the number of participants who accessed the first survey page or consented to participate was not tracked. Only fully completed questionnaires were included in the analysis. |
| **Preventing multiple entries from the same individual** |  |  |
|  | Cookies used | Cookies were not used to assign a unique user identifier. Duplicate entries were limited through survey settings restricting multiple submissions from the same user. |
|  | IP check | IP addresses were not recorded as part of the survey data. Duplicate entries were limited through survey settings restricting multiple submissions from the same user. |
|  | Log file analysis | No additional log file analysis was performed. |
|  | Registration | The survey was conducted in open-access mode without any form of participant registration or login. |
| **Analysis** |  |  |
|  | Handling of incomplete questionnaires | Only fully completed questionnaires were included in the analysis. Incomplete surveys could not be submitted by the participants. |
|  | Questionnaires submitted with an atypical timestamp | No analysis of completion time was performed. |
|  | Statistical correction | No statistical correction methods (e.g., weighting or propensity score adjustment) were applied. |
